# Effect of large dosage of Prunella on Hashimoto's thyroiditis: A protocol of systematic review and meta-analysis of randomized clinical trials

**DOI:** 10.1097/MD.0000000000023391

**Published:** 2020-12-11

**Authors:** Pei Chen, Chaomin Li, Siliang Zhao, Lizhen Wang, Lingyu Liu, Qiuhong Fan

**Affiliations:** Hospital of Chengdu University of Traditional Chinese Medicine, Chengdu, Sichuan, China.

**Keywords:** diabetic nephropathy, large dosage, meta analysis and systematic review, protocol, Prunella

## Abstract

**Introduction:**

Hashimoto's Thyroiditis (HT) is one of the common autoimmune diseases, which can lead to thyroid reduction, increase the risk of tumor, and seriously affect women's reproductive health. Many other autoimmune diseases are easy to occur, seriously harming people's health.large dose herb Prunella or compound prescription contain large dose Prunella for treatment of HT has already been confirmed. However, due to the lack of evidence, there is no specific method or suggestion, it is necessary to carry out a systematic evaluation on Prunella and provide effective evidence for further research.

**Methods and analysis:**

The following databases will be searched from their inception to October 2020: Electronic database includes PubMed, Embase, Cochrane Library, Web of Science, Nature, Science online, Chinese Biomedical Database WangFang, VIP medicine information, and China National Knowledge Infrastructure. Main results: serum thyroid peroxidase antibody (TPOAb), thyroid globulin antibody (TGAb), other results: serum thyroid stimulating hormone (TSH), serum free triiodothyronine (FT3), serum free thyroid hormone (FT4). Data will be extracted by 2 researchers independently, risk of bias of the meta-analysis will be evaluated based on the Cochrane Handbook for Systematic Reviews (SR)of Interventions. All data analysis will be conducted by data statistics software Review Manager V.5.3. and Stata V.12.0.

**Results:**

The results of this study will systematically evaluate the efficacy and safety of large dose prunella salicorrhizae in the intervention of people with HT.

**Conclusion:**

The systematic review of this study will summarize the current published evidence of large dose prunella for the treatment of HT, which can further guide the promotion and application of it.

**Ethics and Communication:**

This study is a systematic review, the outcomes are based on the published evidence, so examination and agreement by the ethics committee are not required in this study. We intend to publish the study results in a journal or conference presentations.

Open Science Fra mework (OSF) registration number:October 21, 2020.osf.io/fcyqp. (https://osf.io/fcyqp)

## 1 Introduction

Hashimoto's thyroiditis (HT) is a common autoimmune disease, characterized by varying degrees of hypothyroidism, elevated thyroid autoantibodies, and lymphocyte infiltration.^[1]^ The prevalence was correlated with age, sex and race.^[2]^ According to statistics, up to 2% of the world's population is affected by this disease, and the incidence of HT in the world is 0.3 to1.5 cases/1000 people every year,^[3]^ while the incidence of HT in China is 0.2% to 1.3%, among which the incidence of HT in women is about 5 to 10 times that in men, showing an increasing trend year by year.^[4]^

HT is an important cause of hypothyroidism. In most HT patients, it is necessary to replace levothyroxine (LT4) for a lifetime and adjust the dose to reach the normal circulating thyrotrophin (TSH) level.^[5]^ However, long-term LT4 substitution therapy will cause lasting damage to the cognitive function of HT patients.^[6]^ In addition, taking LT4 increases the risk of fracture, especially in elderly patients.^[7]^ In addition, HT is easy with a variety of other organ specificity disease [such as pernicious anemia, vitiligo, celiac disease (CD), type 1 diabetes, autoimmune liver disease, primary biliary cirrhosis, myasthenia gravis, alopecia areata, multiple sclerosis, Addison's disease] and non-specific [such as rheumatoid arthritis (RA)], systemic lupus erythematosus (SLE), shed glen syndrome, systemic sclerosis, mixed connective tissue disease), non endocrine autoimmune diseases.^[8]^ In recent years, studies have attempted to treat HT patients with oral vitamin D, selenomethionine, and selenomethionic acid, etc, but their efficacy is limited and confirmation of large sample randomized controlled trials (RCTs) is lacking.^[9–11]^

In recent years, TCM has been widely used in clinical and experimental research of HT, and it has been proved to be effective.Prunella is a botanical herb commonly used in Traditional Chinese medicine. The exploration on the treatment of HT showed that large dose Prunella had a good effect on reducing antibody and improving THE symptoms of HT, but no definite conclusion has been drawn on its efficacy and safety. Therefore, this research intends to adopt the method of system valuation and meta analysis of large-dose Prunella or contain large-dose Prunella prescription in the treatment of HT to evaluate the efficacy and safety.

## 2 Methods

### 2.1 Study registration

The protocol has been registered in OSF(Open Science Framwork) Preregistration.October 21, 2020.osf.io/fcyqp.(https://osf.io/fcyqp). The protocol will follow the statement guidelines of Preferred Reporting Items for Systematic Reviews (SR) and Meta-Analyses Protocols (PRISMAP),^[12]^ Changes will be reported in the full review as required.

### 2.2 Inclusion and exclusion criteria for study selection

#### 2.2.1 Inclusion criteria.

Inclusion criteria are all RCTs, which treatment of HT is large dose Prunella or Prunella is main element in mixture herb formulas. The language of the trials to be included only Chinese or English.

#### 2.2.2 Exclusion criteria. The following courses will be excluded:

(1) patients age < 18 years old
(2) Non-HT.
(3) This therapy is combined with other therapies other than Traditional Chinese medicine.
(4) Non - RCTs and Quasi - RCTs
(5) Case Series and Reviews
(6) Animal research

### 2.3 Types of Participants

Types of participants included people diagnosed with HT, no matter the degree. All the patients should be treated by traditional Chinese medicine included Prunella, or Prunella is main element in mixture herb formulas, or the herb combines with other conventional treatments. No sex, ethnicity, or education restrictions is here.

### 2.4 Experimental interventions.

Prunella vulgaris should be the main treatment in Chinese medicine. The dose limit of these two traditional Chinese medicines should be: large dose of Prunella vulcanis (more than 30 grams, twice the maximum prescription dose of the 2015 edition of the Pharmacopoeia of the People's Republic of China).

### 2.5 Control interventions.

Interventions included no treatment, placebo, non-drug interventions (such as diet, exercise, etc.), and conventional western medicine (levothyroxine).Joint interventions are allowed as long as all groups in a randomized trial receive the same joint intervention.

### 2.6 Types of measurement results

#### 2.6.1 Main results

(1) Serum thyroid peroxidase (TPOAb);
(2) Thyroglobulin antibody (TGAb);

#### 2.6.2 Additional Outciomes

(1) Serum TSH
(2) Serum free triiodothyronine (FT3)
(3) Free thyroid hormone (FT4)

## 3 The data source

### 3.1 Electronic Search

We searched the following databases to identify eligible studies:PubMed, Embase, Cochrane Library, Web of Science, Nature, Science on Line, Wanfang China Biomedical Database, Viper Medical Information, and China's National Knowledge Infrastructure. Time range: The start time is determined according to the first existing literature, and the deadline is October 2020.

### 3.2 Other search resources

**Figure 1:**
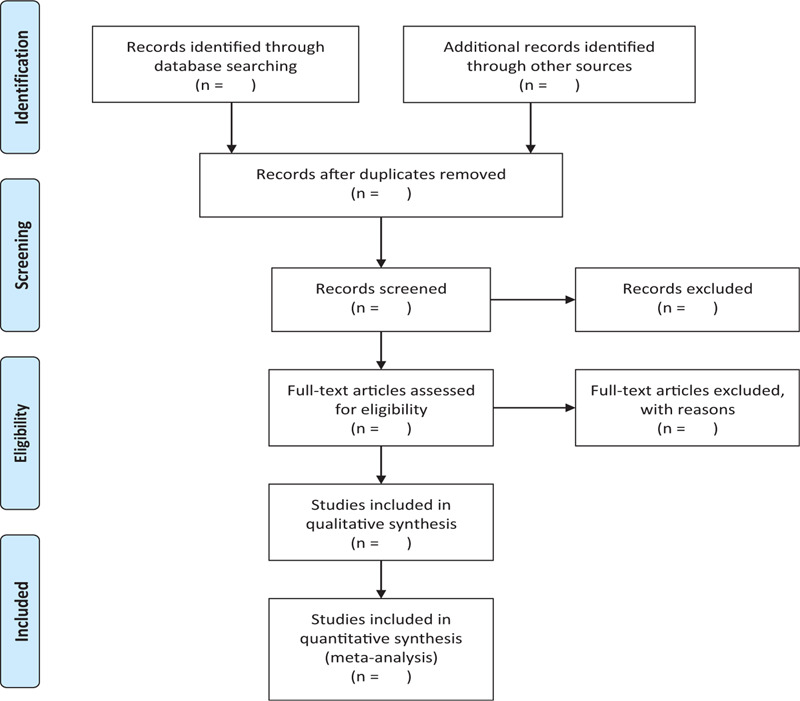
The research flowchart. This figure shows the identification, screening, eligibility and included when we searching articles.

In order to obtain more complete evidence, we will also manually search other relevant literature, such as medical textbooks and clinical laboratory manuals. If necessary, we will contact the trial authors to obtain the latest clinical data. In addition, studies relevant to the review will be identified through evaluation of relevant meeting minutes. The research flowchart is shown in Figure 1.

### 3.3 Search strategy

The following search terms will be used: randomized controlled trial/RCT; HT/HT;Traditional-Chinese-Medicine/TCM; Prunella/Selfheal/Square-stem. Different retrieval strategies in Chinese and foreign databases will be used. Language restrictions are Chinese and English. There is no publication restriction. Here we take the search strategy in PubMed as an example and list in Table 1.

**Table 1:** Search stragtegy sample of PubMed.

number	searchs
1	Hashimoto thyroiditis (MeSh)
2	hashimoto's thyroiditis (ti,ab)
3	Hashimotos thyroiditis (ti,ab)
4	Thyroiditis (ti,ab)
5	Or 1–4
6	Prunella (MeSh)
7	Selfheal (ti,ab)
8	square-stem(ti,ab)
9	prunella spike(ti,ab)
10	selfheal spike(ti,ab)
11	spica prunellae(ti,ab)
12	or# 6–11

## 4 Data collection and analysis

### 4.1 Research Selection

All articles in the search results were independently evaluated by two independent researchers (PC, CL) based on inclusion criteria and exclusion criteria. The evaluator will then independently extract and collect the data included in the study using a pre-designed data collection form. Differences will be discussed and resolved by consensus by the third author (SZ).

### 4.2 Data extraction and management

The following information will be extracted from each study.:1) Normal test features: title, author, year. 2) Baseline data: sample size, age, gender, diagnostic criteria and course of disease. 3) Intervention: Control and intervention of the dosage of Prunella vulgaris and the details of intervention. If the information is insufficient, we will contact experts and authors in the field for relevant information.

### 4.3 Assess report quality and risk of bias

The bias risk was assessed by two independent authors (PC and YQ), who completed the STRICTA checklist.^[13]^ The Cochrane SRs Handbook provides authors with criteria for evaluating the quality of RCTS. Assess the risk of bias:

(1) Random sequence generation;
(2) Allocate and hide;
(3) Blind participants and personnel;
(4) Blindness in result evaluation;
(5) Incomplete result data;
(6) Selective reporting of results;
(7) Other biases.

Any objections will be discussed or consulted by a third reviewer. Each will be described from three levels of “high risk,” “low risk” and “unclear.”

### 4.4 Measures of a treatment effect

Dichotomous results will be represented by odds ratios (ORs), while continuous data will be represented by standardized mean differences. All these outcomes report 95% CIs.

### 4.5 Management of missing data

We will obtain the missing data by contacting the corresponding author. If there is no response, we will only analyze the existing data and describe the causes and effects of this exclusion in the paper.

### 4.6 Assessment of a reporting bias

Publication bias will be explored through funnel plot analysis. If the funnel graph is asymmetric, it will be assessed through Egger and Beg inspection, and *P* value < . 05 indicates significant publication bias.

### 4.7 Evaluation of heterogeneity

There are two main methods for testing heterogeneity, namely graphical method (funnel plot, forest plot) and statistical test (*Q* value statistic test, *I*^2^ statistic test, *H* statistic test). The *I*^2^ statistic test method shows us When *I*^2^ is 0, it means that studies are completely homogeneous, If *I*^2^> 50%, it indicates there is heterogeneity in studies.

### 4.8 Data synthesis and grading of evidence quality

The results will be analyzed using RevMan 5.0 software provided by the Cochrane Collaboration on Network. Binary data are represented by odds ratios and continuous data by mean difference. In order to test the heterogeneity of study results, when *I* < 50% or *P*> .1, the heterogeneity was significant. The random-effect model was used in the meta-analysis; otherwise, the fixed-effect model was selected.

### 4.9 Subgroup analysis

#### 4.9.1 Sensitivity analysis

Sensitivity analysis can not only assess the stability and reliability of the conclusions of the Meta-analysis, but also assess whether the changes in the results are related to the impact of a single study. If the stability of the conclusion is poor, we can achieve when the heterogeneity test results are heterogeneous, we need to clarify the source of the heterogeneity by subgroup analysis. The effects of different types of therapy including design scheme, severity of illness, age, sex, and mild or severe T2DM were analyzed. We will also delete low-quality and/or medium-quality studies to check the robustness of the results.

The purpose of increasing stability by changing the analysis model, inclusion and exclusion criteria, or excluding a certain type of literature.

### 4.10 Morality and communication

We will publish SR results in peer-reviewed journals and disseminate them at conferences or in peer-reviewed publications. Aggregated published data will be used to cull personal data without the need for ethical approval or informed patient consent.

## 5 Discussion

Prunella is a kind of traditional Chinese medicine which can clear the fire, clear the eyes, soften the hard and disperse the knot. Indications: red eye swelling pain, eye bead night pain, headache, dizziness, scrofula, gall, and so on. Modern pharmacological studies have shown that Prunella has anti-tumor, anti-inflammatory, antibacterial, antiviral, blood pressure lowering, hypoglycemic, immune regulation, liver protection, blood circulation and blood stasis removing and other functions. It has been used in a variety of diseases, such as acute and chronic lymphadenitis, lymphatic tuberculosis, simple goiter, as well as breast abscess, mumps and early stage of tumor.^[14,15]^ The main components of Prunella include triterpenoids, sterols, flavonoids, volatile oils, sugars, coumarins, organic acids, and so on.^[16]^ Among them, steroids β - sitosterol (30), stigmasterol

Among them, EAE and NBAE are abundant in total phenolic acids and total flavonoids, and have high antioxidant activities. Therefore, Prunella is a natural antioxidant.^[17]^ In addition, Prunella has significant curative effect in anti-inflammatory and immunosuppressive fields. Cytological studies have also confirmed that abnormal activation of NF - κ B may be closely related to the pathogenesis of HT.^[18]^ Prunella can inhibit the activation of transcription factor NF - κ B.^[19]^ The results of animal experiments showed that in terms of regulating the efficacy of thyroid hormone, pvae could intervene to down regulate the levels of TSH, TG AB, and TPO AB in rat plasma.^[20]^

Traditional Chinese medicine always pays attention to the dosage. According to the Pharmacopoeia of the people's Republic of China (2015 Edition), the standard dose of Prunella is 9 to 15 g, but in clinical practice, Chinese medicine practitioners have their own unique understanding of the specific dose of Poria cocos. The reason why large doses of Chinese medicine can achieve therapeutic effect is that most of them are single traditional Chinese medicine, which usually takes the form of compound or prescription. Clinical experience has confirmed that the dosage of Prunella and its compound preparation is generally large. Song Ning et al. Mainly used Prunella (30 g) orally combined with Zhuang medicine thread point moxibustion in the treatment of nodular acne, the total effective rate was 93.75%.^[21]^ Professor Tong Xiaolin also thinks that Prunella can be adjusted according to clinical practice, and the commonly used dosage is 30 to 60 g.^[22]^ The acute toxicity test and subchronic toxicity test of Prunella showed no obvious toxic effect.^[23]^ Therefore, it is suggested that traditional Chinese medicine rather than chemical extract should be considered in clinical medication, and the dosage should be more in line with clinical practice, so as to accurately reflect the functional value of traditional Chinese medicine and its compound.

In conclusion, SR and meta-analysis are helpful to determine the potential value of large dosage Prunella combined with traditional Chinese medicine in the treatment of HT. To improve the quality of life of critically ill patients. This study can not only provide the basis for the release of HT treatment guidelines, but also promote the application of traditional Chinese medicine prescriptions, so that more patients benefit.

## Author contributions

**Conceptualization:** Pei Chen, Chaomin Li.

**Data curation:** Siliang Zhao, Qiuhong Fan.

**Formal analysis:** Pei Chen, Chaomin Li, Lizhen Wang.

**Methodology:** Pei Chen, Lingyu Liu.

**Project administration:** Chaomin Li, Lizhen Wang.

**Resources:** Pei Chen, Siliang Zhao, Lingyu Liu.

**Software:** Siliang Zhao, Lizhen Wang, Qiuhong Fan.

**Supervision:** Pei Chen, Chaomin Li.

**Writing – original draft:** Pei Chen.

**Writing – review & editing:** Chaomin Li.
